# Heterogeneous Occupancy and Density Estimates of the Pathogenic Fungus *Batrachochytrium dendrobatidis* in Waters of North America

**DOI:** 10.1371/journal.pone.0106790

**Published:** 2014-09-15

**Authors:** Tara Chestnut, Chauncey Anderson, Radu Popa, Andrew R. Blaustein, Mary Voytek, Deanna H. Olson, Julie Kirshtein

**Affiliations:** 1 Oregon State University, Environmental Science Graduate Program, Corvallis, Oregon, United States of America; 2 US Geological Survey, Oregon Water Science Center, Portland, Oregon, United States of America; 3 Biological Sciences, University of Southern California, Los Angeles, California, United States of America; 4 Oregon State University, Department of Integrative Biology, Corvallis, Oregon, United States of America; 5 Astrobiology Program, National Aeronautics and Space Administration Headquarters, Washington DC, United States of America; 6 US Forest Service, Pacific Northwest Research Station, Corvallis, Oregon, United States of America; 7 US Geological Survey, National Research Program, Reston, Virginia, United States of America; Universität Zurich, Switzerland

## Abstract

Biodiversity losses are occurring worldwide due to a combination of stressors. For example, by one estimate, 40% of amphibian species are vulnerable to extinction, and disease is one threat to amphibian populations. The emerging infectious disease chytridiomycosis, caused by the aquatic fungus *Batrachochytrium dendrobatidis* (*Bd*), is a contributor to amphibian declines worldwide. *Bd* research has focused on the dynamics of the pathogen in its amphibian hosts, with little emphasis on investigating the dynamics of free-living *Bd*. Therefore, we investigated patterns of *Bd* occupancy and density in amphibian habitats using occupancy models, powerful tools for estimating site occupancy and detection probability. Occupancy models have been used to investigate diseases where the focus was on pathogen occurrence in the host. We applied occupancy models to investigate free-living *Bd* in North American surface waters to determine *Bd* seasonality, relationships between *Bd* site occupancy and habitat attributes, and probability of detection from water samples as a function of the number of samples, sample volume, and water quality. We also report on the temporal patterns of *Bd* density from a 4-year case study of a *Bd*-positive wetland. We provide evidence that *Bd* occurs in the environment year-round. *Bd* exhibited temporal and spatial heterogeneity in density, but did not exhibit seasonality in occupancy. *Bd* was detected in all months, typically at less than 100 zoospores L^−1^. The highest density observed was ∼3 million zoospores L^−1^. We detected *Bd* in 47% of sites sampled, but estimated that *Bd* occupied 61% of sites, highlighting the importance of accounting for imperfect detection. When *Bd* was present, there was a 95% chance of detecting it with four samples of 600 ml of water or five samples of 60 mL. Our findings provide important baseline information to advance the study of *Bd* disease ecology, and advance our understanding of amphibian exposure to free-living *Bd* in aquatic habitats over time.

## Introduction

Loss of biodiversity in terrestrial, freshwater, and marine systems is occurring at a global scale [1]–[5]. The causes of losses are often complex and include synergistic effects of natural and human-induced stressors, such as habitat loss and fragmentation [6], [7], urbanization [8], invasive species [7], [9], [10], contaminants [11]–[13], global climate change [14]–[16], and emerging infectious diseases [17], [18]. In the last 35 years, one estimate suggests that the amphibian extinction rate is at least 105 times higher than the expected rate [19], with 32.5 to 41% of amphibian species threatened [7], [20]. In the USA, the number of sites occupied by amphibians is declining by an estimated 3.7% per year [20], [21].

Among the many threats to amphibians, the role of disease in population declines has been recognized increasingly over the last two decades. Numerous amphibian diseases have been identified, with mass mortality events attributed to water molds (*Saprolegnia* spp.) [22]–[24], *Aeromonas* bacterial infections [25], iridoviruses [26]–[29], alveolate infections [30], [31], and malformations caused by trematodes [32], [33]. Chytridiomycosis, the emerging infectious disease caused by the amphibian chytrid fungus, *Batrachochytrium dendrobatidis* (*Bd*), is implicated as a causal agent in many recent global amphibian population declines and extinctions [34]–[36].

Of the over 3,000 fungal species described from aquatic habitats [37], chytrid fungi are the earliest of extant fungi to diverge phylogenetically, and now have a global distribution [38], [39]. *Bd* is one of over 1,200 chytrid species described from freshwater, marine, and terrestrial systems occurring across temperate, tropical, and tundra environments [40]–[42]. Although chytrids function primarily as plant saprobes and parasites [37], some also parasitize animals [43], [44]. *Bd* is one of only two chytrids known to infect vertebrate hosts [44], [45].

To understand the pathology and conservation implications of *Bd*, a better understanding of its distribution, ecology, and the life history of *Bd* in the wild is needed. The life cycle of *Bd* includes forms that are free-living in the aquatic environment [46], [47]. *Bd* has been detected by filtering water samples to capture free-living zoospores and zoosporangia, then performing a genetic analysis on the filtered particulates [48]–[50]. *Bd* has not been reliably detected from sediments [48]. Laboratory experiments have demonstrated that *Bd* has survived on sterilized moist sand for up to three months and remained infective in lake water for up to seven weeks [51]. *Bd* cultures can be maintained under laboratory conditions for several years (TC, ARB personal observation), which suggests that *Bd* can survive in the environment without a host as long as conditions are favorable. In laboratory settings, *Bd* growth and reproduction depended on temperature (4–25°C, ideal 17–23°C) and pH (4–10, 6–7 ideal) [52], and differences in both generation time and fecundity of *Bd* in response to different thermal regimes are observed in at least one *Bd* strain [53].

A greater understanding of *Bd* distribution and spread is of paramount importance to more fully understand its threat to amphibians [36]. Although it has an impact on the persistence of selected amphibian populations around the world [21], [35], [54], the full scope of the effects of chytridiomycosis on global amphibian population declines is not well understood. Most *Bd* research efforts have focused on *Bd* in amphibian hosts *per se*, with little focus on the ecology of free-living *Bd* outside of the amphibian host [55]–[57]. In amphibian hosts, *Bd* exhibits sensitivity to a number of environmental variables including temperature [36], [58]–[61]and elevation [59], [62], suggesting a tendency to associate high-elevation areas or regions with cool temperatures with increased risk for *Bd-*related declines and extinctions [63]. From a large global data set of amphibians sampled for *Bd* infections, it was found that *Bd* occurrences were inversely correlated with the temperature range at sites [36]. Seasonal patterns of amphibians infected with *Bd* have emerged in several studies [58], [64]–[67], further implicating microclimatic associations and potential differences between strains [68]. In wild amphibian populations from temperate areas, *Bd* infections appear to follow predictable patterns, with the highest prevalence and intensity in the cooler spring months, and decreasing prevalence in the warmer summer and autumn months, sometimes to non-detectable limits [66], [67]. We describe spatial and temporal patterns in occupancy and density of free-living *Bd* in aquatic habitats of the United States, outside of the amphibian host, to better understand the geographic distribution and ecology of this pathogen, and to further inform pathogen dynamics research.

**Figure 1:**
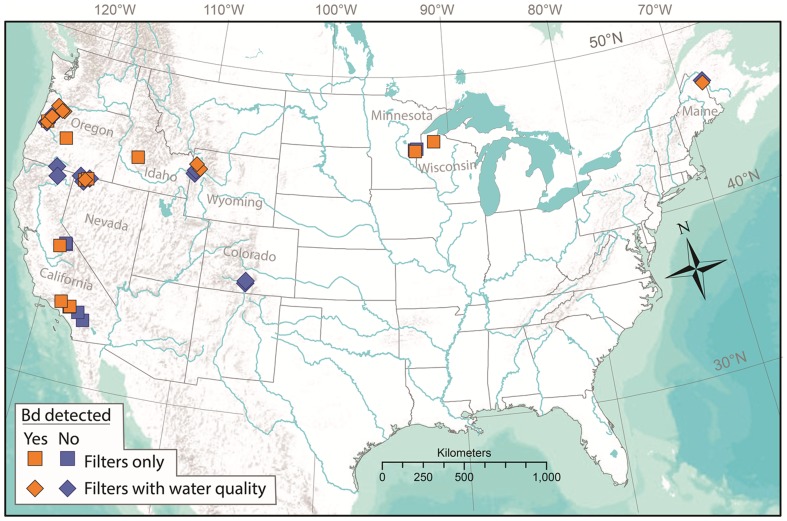
Sampling locations for *Batrachochytrium dendrobatidis* (*Bd*) in temperate North America with results of *Bd* detections from water samples.

Occupancy modeling is a powerful tool to estimate site occupancy and detection probability when detection is imperfect [69]. Occupancy models have been used to investigate disease ecology in amphibians, fishes, and birds, where the focus was on pathogen occurrence in the host [70]–[72]. We used an occupancy modeling approach to investigate free-living *Bd* in North American surface waters, from water samples collected across several sites in North America (Figure 1) to assess: 1) the seasonality, if any, of *Bd* detection; 2) the relationships between *Bd* site occupancy and habitat attributes including water quality (temperature, pH, specific conductance), geography (latitude, longitude, elevation), climate metrics (minimum/maximum/range in precipitation and temperature, dew point), and time (Julian day); and 3) the probability of *Bd* detection from water samples as a function of the number of samples taken, sample volume, and water quality (temperature, pH, specific conductance). We predicted that free-living *Bd* occupancy and density would follow the same trends as observed in *Bd*-infected amphibians, with the highest detections in the North American spring season and the lowest in the late summer/autumn [64], [66], [71]. Based on reported *Bd*-amphibian host relationships and laboratory studies of *Bd*, we expected to see corresponding temperature and pH associations in our analyses. We also predicted that detection probability would increase as the number of samples and volume of water per sample increased, and would increase as temperature and pH approached the ideal range for *Bd* growth [52]. Finally, we report on the temporal patterns of *Bd* density from a four-year case study of a *Bd*-positive wetland, and the relationship between water quality and *Bd* density.

Our findings address important baseline information to advance the study of *Bd* disease ecology in temperate-zone systems, and specifically to advance our understanding of the likelihood of amphibian exposure to free-living *Bd* in aquatic habitats over time. Our results are applicable to *Bd* risk assessment, sampling protocols, policy development, and regulatory decision-making processes.

## Methods

### Ethics Statement

Field studies involved collecting water samples. No animals were sampled as part of this study. Sampling did not involve endangered or protected species, although they may have occurred at or near the sites sampled. Permission to access National Park Service lands was obtained by Bill Commins, NPS Research Permit Coordinator, Washington, DC (bill_commins@nps.gov). Private lands were accessed with landowner permission; TC may be contacted for future permissions. Public right-of-ways did not require permission/permits to sample. Given the sensitive nature of some sites, GPS coordinates are available upon request.

### Field Methods

#### All Sites

We collected *Bd* water samples from 41 amphibian breeding sites along a latitudinal gradient spanning the North American continent between 45 and 48 degrees North, and a longitudinal gradient focused on, but not exclusive to, the western United States west of 105 degrees West (Figure 1). Site selection occurred opportunistically at locations where on-going amphibian research was being conducted; hence our scope of inference is restricted to the sampled sites. Sites were located in temperate ecoregions in a variety of land-use settings ranging from urban/suburban to designated wilderness, and at elevations ranging from near sea level to ∼3500 m—39% were low-elevation, 54% were mid-elevation, and 7% were high-elevation. Three replicate water samples were collected, separated by >50 m, during a single survey occasion that occurred between May and September, 2007 to 2010. Most sites (63%) were sampled in June and July. We measured water quality (i.e., temperature, pH, specific conductance) at 22 of 41 sites, herein WQ sites (Figure 1).

#### Case Study Site (Beaverton, Oregon)

We also conducted a four-year case study in Beaverton, Oregon (45.3108, −122.4752, Datum: WGS84), which we monitored monthly between 2007 and 2011 to assess temporal variation in *Bd* occurrence and density. The site was a palustrine wetland in a suburban setting, dominated by reed canary grass (*Phalaris arundinacea*). It was approximately 1.2 hectares in area, bisected by a road, and connected to larger wetland complexes 6.4 hectares in area associated with Johnson Creek, both upstream and downstream of the site. Surrounding land use was historically agriculture (row crops and horse pasture), and is now primarily residential. In the 1990s the site was converted to a wetland mitigation site and has undergone recent efforts to reestablish a forested wetland.

Water samples were collected using the protocol described in [48], with modifications to improve *Bd* DNA recovery and sampling efficiency, which are described below. At each site, three spatial replicate water samples were collected for *Bd* testing, also at least 50 m apart in the pond or wetland. Each sample was collected from shallow water, between 5 and 20 cm below the water surface. A general overview of sample collection is as follows: using a sterile 60-mL syringe rinsed 3 times with native water, we filtered water drawn from below the surface through a Sterivex 0.22-µm capsule filter (luer-loc with male end) until the filter was nearly clogged. The volume was recorded (range: between 20 mL and 2.4 L, mean 350 mL) and the filter was flushed using a new sterile syringe with 50 mL of DNAase/RNAse-free 0.01 Mol Phosphate Buffered Saline (PBS) to remove excess dissolved organic carbon which can inhibit the PCR reaction. The filter was purged of water by removing it from the syringe, drawing air into the syringe, reattaching the filter and pushing air through the filter and repeating if necessary. The outflow end of the capsule filter was sealed with Hemato-Seal clay (we found that other types dried and cracked in the field, compromising the seal), then we injected the filter with 0.9 mL cell lysis buffer solution, and sealed it with a luer-loc cap. Each sample was placed in an individual Whirl-pak bag and kept cool. Upon returning from the field, samples were refrigerated until DNA extraction occurred, within three months of sample collection. Water quality was measured with a single YSI multiparameter data sonde, calibrated before each sampling event using standardized U.S. Geological Survey (USGS) protocols [73]. The YSI was cleaned between each use.

The method we used to collect *Bd* samples involves water filtration and molecular techniques but differs from what is usually termed environmental DNA (eDNA) sampling [74], [75]. Typical eDNA methods sample the environment for DNA from parts of an organism (e.g., shed skin, tissue fragments), whereas the method we used is a standard method for characterizing microbial communities, where whole microbes are captured on the filter and dissolved DNA is passed through the filter. This ensured we were sampling only *Bd* that occupied the site at the time the sample was collected. However, it is possible that if we filtered recently shed amphibian skin infected with *Bd*, it may have been captured by our water sampling process.

### Laboratory Methods

DNA was extracted from the cell lysis solution using the Gentra Puregene Tissue Kit [48]. We added proteinase K (0.1 mg mL^−1^) directly to the filter by opening the luer-loc cap, injecting the solution and resealing the capsule. We placed each filter in a sterile 50-mL Falcon tube, and incubated it at 55°C for 60 min in a continuously rotating rack inside the incubator to ensure the filter was completely bathed in the lysis/proteinase K solution. Following this step, we drew the solution out of the filter with a sterile syringe, and continued the extraction following the manufacturer's protocol. Quantitative PCR assays were conducted using the Qiagen QuantiTect SYBR Green PCR kit following standard protocols [48]. To quantify the *Bd* zoospore equivalents, we calculated a conversion factor from a dilution series of known zoospore numbers (i.e., 169), which is within the range of 60 to 220 for rRNA fungal gene locus copy numbers [76]. *Bd* genomic equivalents are therefore 169 times greater than the numbers we report. A site was considered *Bd*-positive if at least one of three replicate filters from a site returned a positive qPCR result.

### Statistical Methods

Occupancy models require multiple observations per site. We treated each water sample as an independent observation, for a total of 3 observations per site. We used a single-season occupancy model to simultaneously estimate the site-level probability of *Bd* occurrence in amphibian habitats (Ψ) while accounting for the observation-level probability of detecting *Bd* in amphibian habitats when it is present (p), and relating both parameters to our hypothesized covariates. We investigated eight covariates that we hypothesized were related to the probability of occurrence (Ψ) and five covariates we hypothesized were related to detection probability (p) in amphibian habitats, and built models for all possible combinations of the a priori covariates (Table 1). Temporal (day of year) and geographic (latitude, longitude, and elevation) predictors of occupancy were recorded at the time of sampling. Other environmental predictors of occupancy (precipitation, minimum temperature, maximum temperature, temperature range) were derived from PRISM (Parameter-elevation Regressions on Independent Slopes Model) climate mapping system data at a spatial resolution of 1 km^2^ [77]. Two covariates of p were recorded from all sites (p as a function of volume, and p as constant across surveys), and three covariates of water quality (temperature, pH, specific conductance) were recorded from the subset of 22 WQ sites. Continuous covariates were standardized to mean = 0 and standard deviation = 1. We evaluated the degree of support for each competing model based on Akaike's Information Criteria (AIC) and the resulting Akaike's weights. We considered that models within 2 delta AIC received similar degree of support. Using the maximum likelihood estimates from the top-performing model for all 41 sites, we also estimated the detection probability given the number of samples collected and the volume of the water filtered per sample. In the subset of WQ sites, we further investigated three water quality covariates (temperature, pH, and specific conductance) that potentially related to the probability of *Bd* detection, using the top-performing detection probability models (Table 2). In the Oregon case study, we examined associations between *Bd* detection and four water quality metrics (temperature, pH, specific conductance, and turbidity) using Pearson's correlation coefficient. To include this site in our occupancy analysis, we randomly selected one sampling event that occurred within the sampling period of the other sites, (between May and September, and between the years 2007 and 2010). Data were summarized using the base packages available in R 2.14.0 [78]. To examine the relationships between *Bd* occupancy of amphibian habitats and our covariates of interest, we developed models using the software Presence 4.4 (http://www.mbr-pwrc.usgs.gov/software/presence.html). *Bd* data are archived in the USGS Western Ecological Research Center Multitaxa Database and water quality data are archived in the USGS National Water Information System.

**Table 1:** Covariates hypothesized to relate to the probability of detection (p) or occurrence (Ψ) of *Batrachochytrium dendrobatidis* (*Bd*) in amphibian habitats, including data source and model fit.

Covariate abbreviation and description	Probability of occurrence (Ψ)	Detection probability (p)	Data source	Included in best fitting models
vol = volume of water filtered from an amphibian habitat to the nearest mL	-	X	Field measurement	-
temp = temperature in degrees Celsius to the nearest tenth at the location a sample was collected	-	X	Field measurement	-
ph = pH at the location a sample was collected	-	X	Field measurement	X
sp_cond = Specific conductance in microsiemens (uS), standardized to 25°C, at the location a sample was collected	-	X	Field Measurement	-
lat = latitude in decimal degrees, datum WSG84	X	-	field measurement, GIS verified	-
long = longitude in decimal degrees, datum WGS84	X	-	field measurement, GIS verified	-
elev = elevation in m to the nearest whole number	X	-	field measurement, GIS verified	X
precip = precipitation in mm in the 24 hours prior to sampling	X	-	PRISM data (Daly et al. 2008)	x
tmin = minimum temperature in degrees C in the 24 hours prior to sampling	X	-	PRISM data (Daly et al. 2008)	-
tmax = maximum temperature in degrees C in the 24 hours prior to sampling	X	-	PRISM data (Daly et al. 2008)	-
Trange = temperature range in degrees C in the 24 hours prior to sampling	X	-	PRISM data (Daly et al. 2008)	x
day = day of year	X	-	calculated value	-

In our exploratory analysis, we built models for all possible combinations of the a priori covariates and eliminated covariates that had low predictive power. X indicates the best fitting model, based on AIC value and weight; x indicates a covariate identified in a model with a delta AIC value of <2 but less support as indicated by AIC weight.

**Table 2:** Model selection statistics for a priori models relating to occupancy of *Batrachochytrium dendrobatidis* (*Bd*) from 41 amphibian habitats with environmental and geographic covariates in temperate North America, where detection probability is constant p(.), as differences in detection probability were negligible when the pH range was considered.

Model	AIC	deltaAIC	AIC wgt	Model likelihood	# of parameters	.-2*LogLike
psi(elev),p(.)	139.89	0	0.2284	1	3	133.89
psi(elev+precip),p(.)	141.3	1.41	0.1128	0.4941	4	133.3
psi(elev+Trange),p(.)	141.61	1.72	0.0966	0.4232	4	133.61
psi(elev+lat),p(.)	141.82	1.93	0.087	0.381	4	133.82

## Results

**Figure 2:**
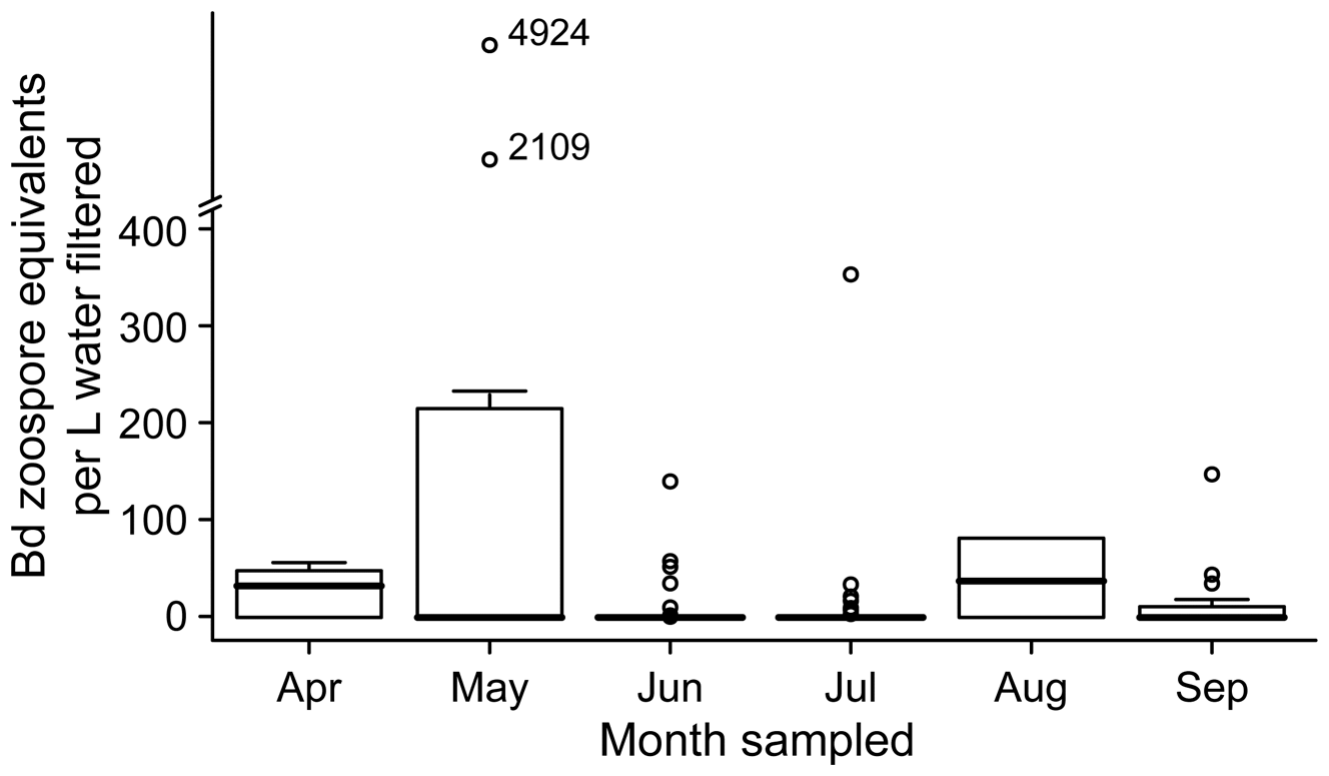
*Batrachochytrium dendrobatidis* (*Bd*) zoospore density from 41 amphibian survey sites measured between April and September, 2007 to 2010.

**Figure 3:**
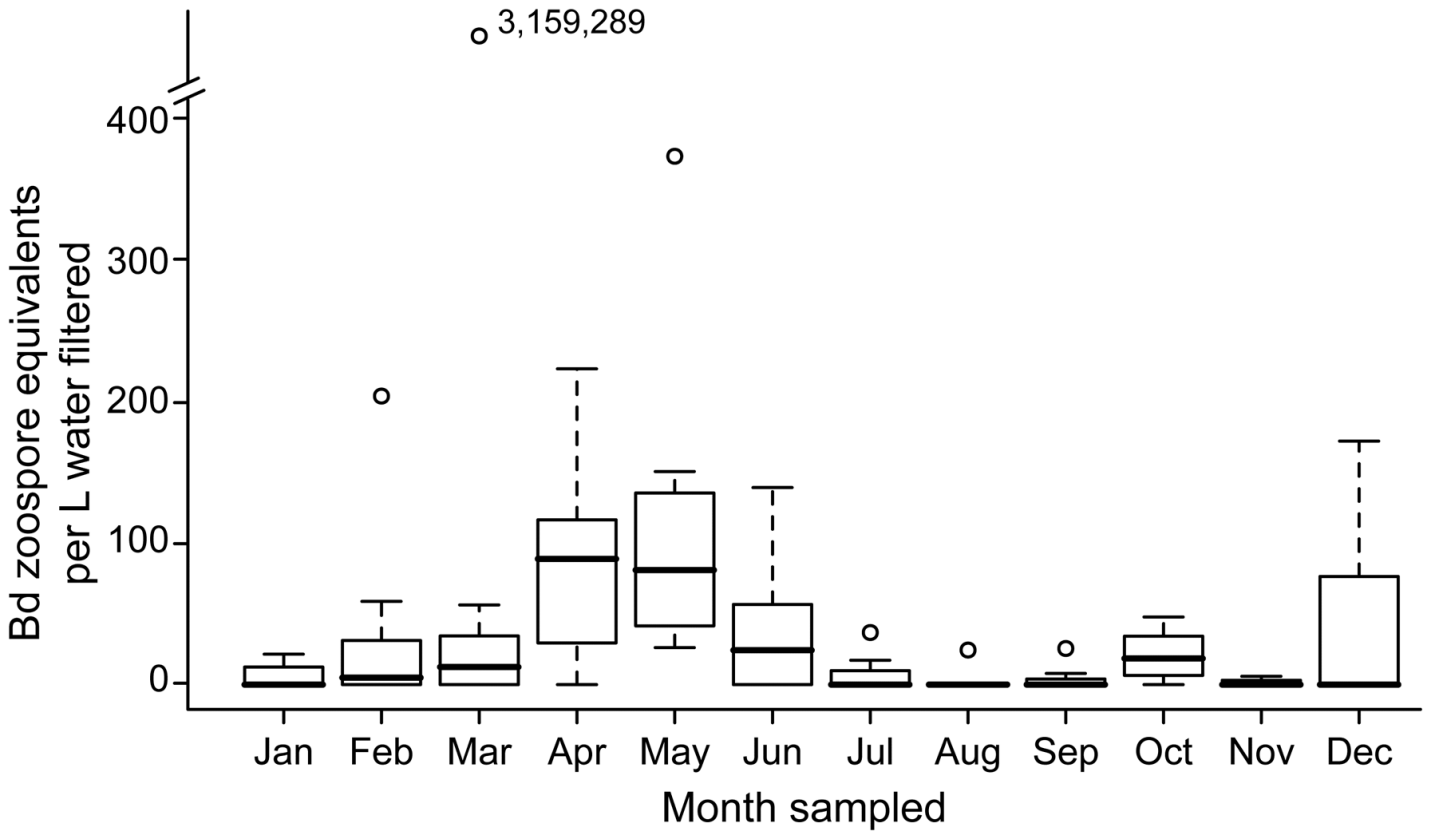
*Batrachochytrium dendrobatidis* (*Bd*) zoospore density from a four year case study site in Oregon, July 2007-March 2011.

**Figure 4:**
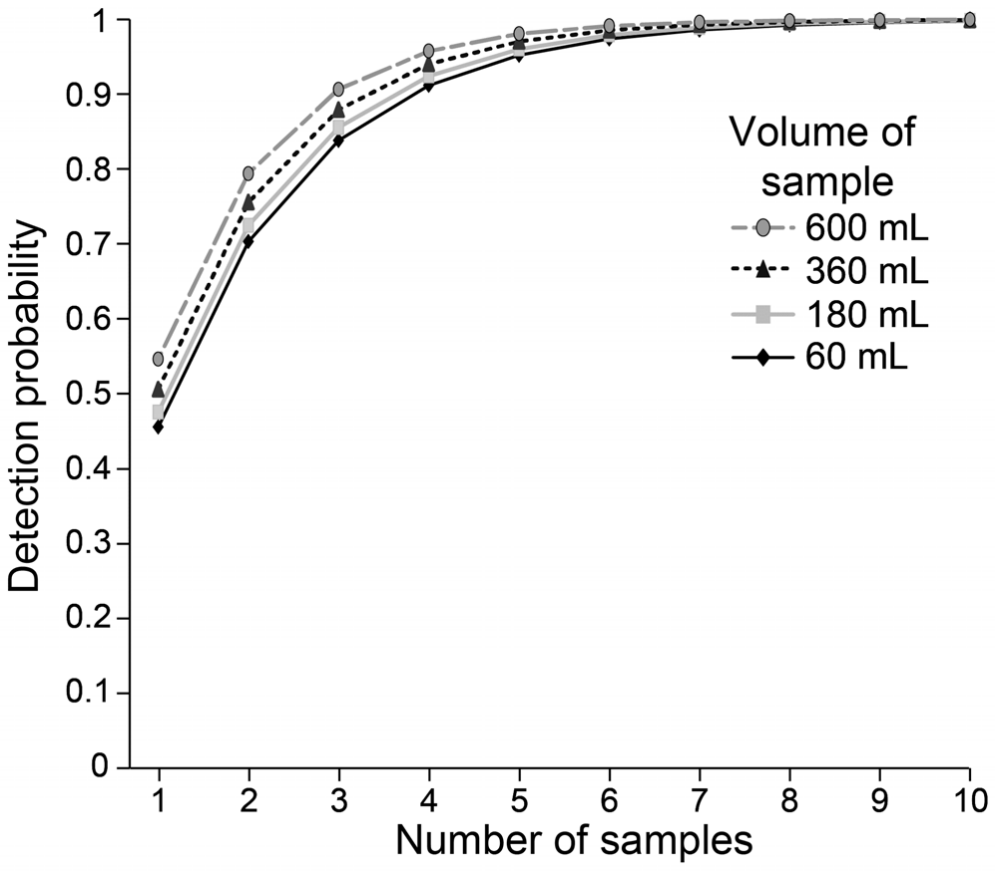
Variation in detection probability of *Batrachochytrium dendrobatidis* (*Bd*) in water collected from amphibian habitats (vol = 60–600 mL/sample) as a function of (i) the number and (ii) the volume of water samples collected.

*Bd* exhibited temporal and spatial heterogeneity in detection and density in amphibian habitats. In both our landscape-scale study of North America and the Beaverton, Oregon case study we detected *Bd* in all months surveyed, but not in all samples. *Bd* density was highest in spring (Figures 2 & 3), and at two Nevada sites sampled in May, *Bd* densities were more than 50 times the densities that we observed at other sites (2,109 and 4,924 zoospores L^−1^). At all sites, mean zoospore densities were highest in April and August (Figure 2, overall mean: 40 zoospores L^−1^; median: 15 zoospores L^−1^; range: 0–4,924 zoospores L^−1^.). In some cases, we observed marked heterogeneity between the individual samples, e.g., at the two Nevada sites, one sample recovered 2,109 zoospores L^−1^ from the wetland and the other two filters recovered an order of magnitude less, 192 and 227 zoospores L^−1^. At the site where 4,924 zoospores L^−1^ were recovered, *Bd* was not detected from the other two filter samples. Our Oregon case study had a higher mean density than observed in our landscape-scale study (i.e., 100 zoospores L^−1^) and the highest *Bd* density observed from a field collected sample, 3,159,289 zoospores L^−1^ (Figure 3). There was a 95% chance of detecting *Bd* at a site when it was present with 4 samples of 600 mL of water or 5 samples of 60 mL of water (Figure 4), estimated by calculating 1–(1-p)∧#samples.

### All Sites

**Figure 5:**
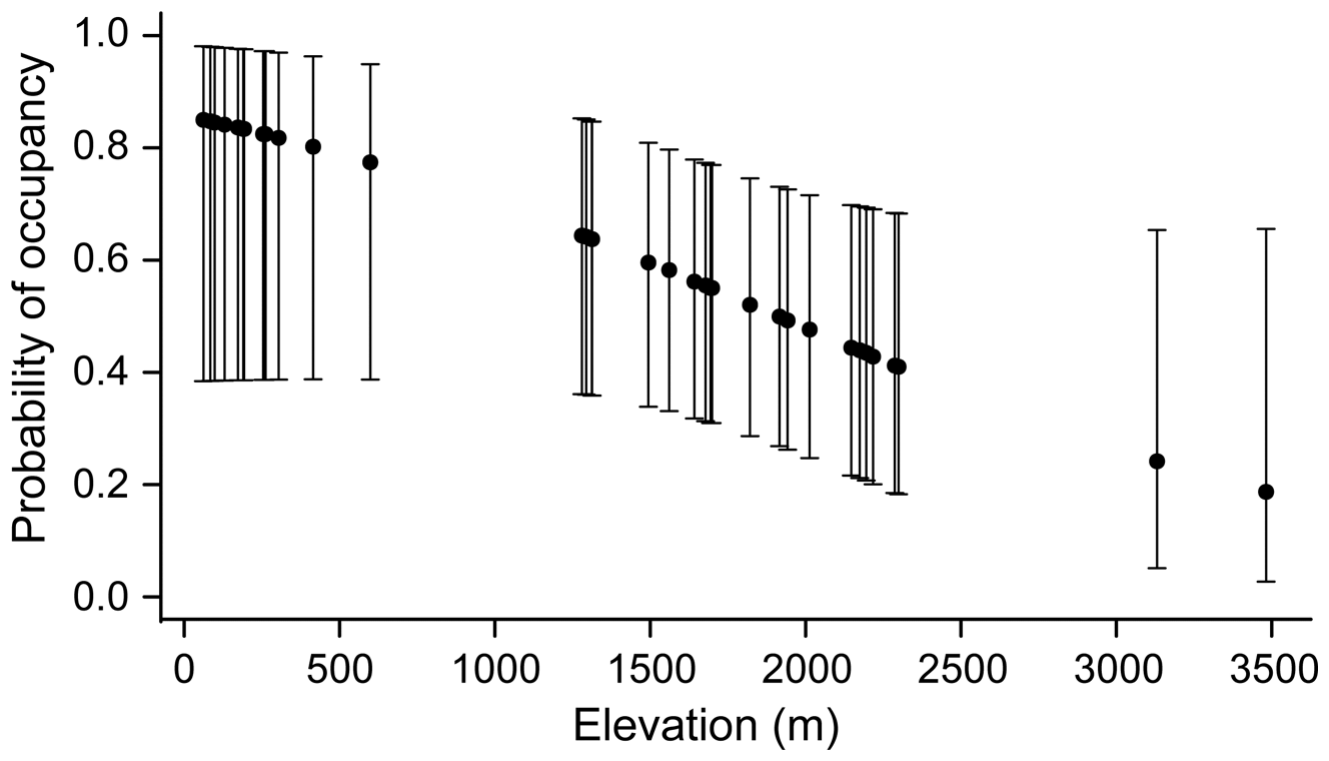
Variation in detection probability of *Batrachochytrium dendrobatidis* (*Bd*) in water collected from amphibian habitats *Batrachochytrium dendrobatidis* (*Bd*) as a function of elevation in meters.

**Figure 6:**
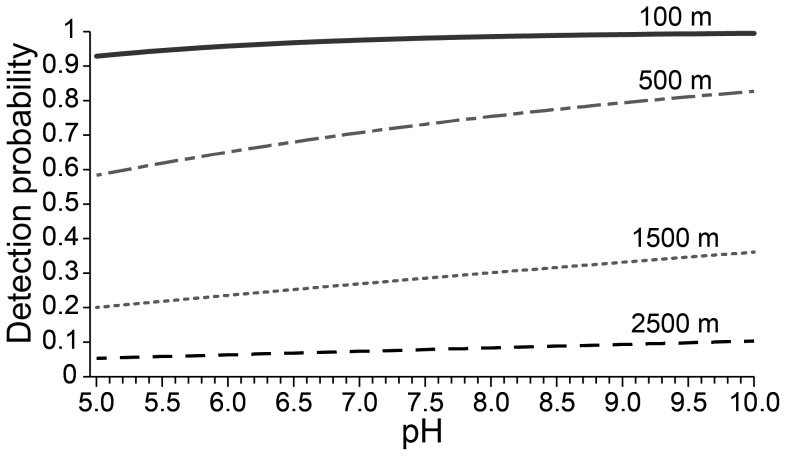
The probability of detecting *Batrachochytrium dendrobatidis* (*Bd*) in water samples at four elevations within a range of pH.

We detected *Bd* in 19 of 41 (47%) of sites sampled, however our occupancy estimate was much higher, suggesting 61% of amphibian habitats were occupied by *Bd* (SE = 11%, CI = 39%–80%), underestimating *Bd* in our study by 14%. Models with elevation were the best fit to predict *Bd* occupancy (Table 2); as elevation decreased, *Bd* occupancy increased (Figure 5). At 100 m elevation, the probability of detecting *Bd* when it was present was between 92–99%. At 500 m elevation, the probability of detection decreased slightly to 81–96%. However, at higher elevations, detection probability decreased sharply, 36–59% at 1500 m, and only 10–20% at 2500 m. The best-supported model structure for parameter ‘p’ was p(pH); the probability of detecting *Bd*, when present, increased as pH increased, however differences in detection probability were negligible when the pH range was considered (Figure 6). Observed pH ranged from 6.5 to 10.4 (mean = 7.8, median = 7.6). *Bd* was detected in samples with a pH between 6.5 and 8.5 (mean = 7.1, median = 7.1, n = 14 samples from 8 sites). Because there was a negligible difference between the p(ph) model and the p(.) model (ΔAIC = 1.1), we proceeded with the most parsimonious model for estimates of Ψ (Table 2).

### Case Study Site (Beaverton, Oregon)

Across all years, we detected *Bd* at the Oregon site at least once per month, although not necessarily in every month in a given year (e.g., *Bd* was detected in December during 2 of 4 years sampled). As predicted, *Bd* zoospore density was highest in the spring, March to June, and contrary to our landscape-scale analysis, lowest in summer (Figure 3). Mean *Bd* density was similar in March and October, and an unexpected peak was observed in December in 2 of 4 years (2007 and 2008). From one sampling event in March 2011, we recovered 3,159,289 zoospores L^−1^ from one filter, approximately four orders of magnitude larger than any previous observation in our study (e.g., Nevada sites mentioned above). The *Bd* densities from the other two filters from this March 2011 sampling event were 0 and 4 zoospores L^−1^. *Bd* detection and density at the Oregon site were not correlated with any of the water quality variables that we measured (temperature R^2^ = 0.01, pH R^2^ = 0.02, specific conductance R^2^ = 0.02 and turbidity R^2^ = 0.02).

## Discussion

*Bd* is emerging as an extremely challenging pathogen to fully comprehend, and our baseline information of the temporal and spatial patterns of *Bd* occurrence in USA waters, free-living, apart from amphibian hosts, adds a new dimension to understanding the dynamics of the pathogen. Host-pathogen systems are more complex when a pathogen can infect multiple host species rather than a single species [79]. Unlike most chytrid parasites, *Bd* is a generalist, infecting at least 42% of the species of amphibians examined [36]. Globally, *Bd* occurrence in amphibians increased as species richness increased, although in North America when species richness was <10, as was the case in our study area, the odds of *Bd* detection were constant [36]. *Bd* detection probability in water also increased as amphibian density at a site increased [80]. This may be due to an increase in the number of competent hosts, direct skin contact with infected individuals, or exposure to free-living *Bd*. Also, *Bd*-positive amphibians arriving at breeding sites from upland overwintering sites may release *Bd* into the aquatic environment, causing an increase in *Bd* density in the water. Alternatively (or concurrently), spring-season conditions (e.g., increasing temperature and light) and amphibian arrival at a breeding site may stimulate free-living *Bd* to grow in the aquatic environment. Adding to the complexity is context-dependency, where different species, life stages, and populations of amphibians may respond to a pathogen such as *Bd* differently, depending on biotic and abiotic factors acting in concert with each other, including strain differences and variation in organismal responses between sites [81]–[83].

*Bd* did not exhibit seasonality in occupancy (e.g., presence) in our study. We detected *Bd* in all months, which is evidence that *Bd* persists in the environment year-round in sites in the U.S. In amphibians within the geographic scope of our study, *Bd* has been estimated to occur in 53% of amphibian populations sampled, and in 66% of non-native bullfrog (*Lithobates catesbeianus*) populations sampled [71], estimates that are similar to our occupancy estimates for *Bd* in amphibian habitats (61%). Areas for future research include analyses of: (i) the substrates upon which *Bd* lives in the environment in the cold season when amphibian densities at the ponds are low or zero; (ii) spatial heterogeneity within a wetland or pond; (iii) alternative hosts, vectors, and reservoirs in different seasons; (iv) *Bd* strain diversity, especially in areas with introduced species; and (v) pathogenicity, or infectious potential, of *Bd* when amphibians are absent for months or years.

The occurrence of a species and its abundance are influenced by habitat factors at different temporal and spatial scales, from microhabitat to landscape [84]–[89]. In temperate areas, there is seasonal variation in *Bd* infection prevalence (i.e., the proportion of a population infected) in amphibian populations, with the highest prevalence in the early spring and lowest in the autumn [66], [90]. Our study calls attention to the heterogeneity that may exist within a site and across the landscape over space and time. We observed a clear signal of increased *Bd* density in the spring. Our results were consistent with our prediction that *Bd* density in water would be high in the spring, and contrary to our prediction that *Bd* density would be low in the summer. The effect of season may be driven by various factors; the temperatures during the spring months are within the range for optimal *Bd* growth [52], and *Bd*-infected animals are returning to breeding sites, shedding zoospores into the water and transmitting the infection to each other as they congregate. Alternatively or additionally, springtime increases in host abundance may elicit a chemotactic response from free-living *Bd* [91], which could contribute to an observed increase in the zoospore density in water.

Adding complexity to the effort of understanding *Bd*'s temporal dynamics in prevalence, alternative *Bd* hosts and reservoirs need to be ascertained, and their potential interactive role in amphibian-*Bd* systems is only beginning to be explored. Several documented and suggested alternative *Bd* hosts and reservoirs (e.g., crayfish [92]; nematodes [93]; birds [94], [95]) occur within the same water bodies as amphibians, and some potential host/reservoir species (e.g., zooplankton) consume chytrid spores, including *Bd* [43], [96], although their post-consumption fate is unknown. When consumed, up to 50% of parasitic fungal spores may survive passage through the gut, which may further fuel disease epidemics [97]. However, there is experimental evidence that consumption by zooplankton can negatively affect free-living *Bd* such that infections in tadpoles are reduced [98], which suggests the aquatic-community context is an important consideration with regard to disease dynamics. Furthermore, *Bd* can survive in pond water for months without a host [51], and occurrence in benthic sediments appears to be likely, although detection is inhibited, perhaps due to high levels of organic carbon inherent in sediment samples from these habitats [48]. These combined factors make interpreting the *Bd*-amphibian disease system in a field-based setting particularly challenging, and highlight the need for a more focused effort to study *Bd* life history in the aquatic environment.

In our study, *Bd* exhibited seasonality in density, but the pattern depended on the spatial scale considered (North American landscape-scale or single-site case study). At the landscape level, the mean densities observed did not follow all of the patterns we predicted. While we did observe high densities in spring months, we also observed high densities in at least one of the summer months, August. This may have been a limitation of our study design; if sites had been visited more than once, we could have made comparisons of relative densities between visits. At our Oregon case-study site, we observed the predicted temporal patterns, with exceptions. We repeatedly observed similar mean densities in late spring and late autumn (March and October), as well as a secondary peak in winter (December), when we expected *Bd* density to be low. There were no major storm or flood events in the days prior to our sampling that would have flushed or resuspended *Bd* or potential physical reservoirs such as sediments into the wetland. Our observations lead to more questions about the role of potential secondary host species and reservoirs in regulating *Bd* densities in amphibian habitats. Crayfish are identified as a potential secondary host [92], but we did not observe them at the Oregon case-study site in our four-year monitoring period. Although it was a single observation, our sample of over 3 million zoospore equivalents from a liter of water is notable because it was several orders of magnitude higher than any other sample collected (the sample was run multiple times to ensure no lab errors). The sample was collected in March 2011, during the predicted spring peak, and it is possible that we captured a large free-floating colony of zoosporangia. Such colonies might be expected to be patchy in distribution, and our sample may have haphazardly collected such a localized high-density cluster. This observation strongly suggests that *Bd* zoospores can have a patchy/aggregated distribution and highlights the importance of collecting several spatial replicates from a pond or wetland during water sampling. The observed temporal variation in our study is intriguing, and suggests an interplay of abiotic and biotic factors, highlighting the relevance of finer-scale assessments. Our results demonstrate that we can correct for this clustering and reliably detect *Bd* by adjusting the number of samples and volume of water collected. Investigations of the patterns and processes influencing *Bd* occupancy in amphibian habitats may be most appropriate at an ecoregional or local scale [21], [36]. Factors influencing detection probability may be site-specific and context-dependent [66], [71]. Importantly, such studies can be informed by both landscape approaches (like ours), and insights from laboratory experiments (e.g., investigating primary and secondary hosts, reservoir species, and amphibian species and life stage) that can shed light on local, regional, and global amphibian declines [83], [92], [98].

### Elevation as a predictor of occupancy

The fact that *Bd* occupancy within our study area was highest at low-elevation sites suggests that distribution patterns and geographic range may be influenced by human-induced landscape change, or differences in host-species composition. The probability of detecting *Bd* when it is present increases as the Human Footprint, an index human activities, increases (i.e., human population size, density of secondary roads, proportion of agricultural lands) [71]. This generalized index doesn't identify specific human activities, but provides a direction for future studies of ecophysiology, or the links between environmental stress, endocrine-immune interactions, and disease [99]. Bullfrogs are a common non-native species implicated as disease vectors throughout our study area at lower elevations that are absent from higher elevations, although they demonstrate differential responses to North American versus Globally Distributed *Bd* strains [68]. The proportion of sites occupied by *Bd* is higher when non-native bullfrogs are present (66%, versus 53% when bullfrogs were absent), which may explain why we observed higher occupancy as elevation decreased, though bullfrog presence alone does not explain widespread *Bd* occupancy of amphibian habitats [71], [100].

Although temperature was not a predictor of *Bd* occupancy, the temperature at high elevations often has a greater range than at lower elevations. Temperature influences both generation time and fecundity of *Bd*, and differences in long-term responses to different thermal regimes are observed in at least one *Bd* strain [53], [57]. Cold-adapted *Bd* develops faster than warm-adapted *Bd*, however, overall developmental time is longer; it may take 4 times longer to encyst, and take 6 times longer to mature and release zoospores [53]. These factors may explain differences in occupancy between our high- and low-elevation sites, and they could influence detection probability if *Bd* density is lower as a result of decreased generation time or fecundity.

At a global scale, there are family-level differences in the odds of *Bd* infection related to elevation. Elevation was found to be (i) positively correlated with Bd detection/infection in toads (Bufonidae); (ii) negatively correlated with *Bd* detection in true frogs (Ranidae); and (iii) not a predictor for *Bd* detection in treefrogs (Hylidae) [36]. However, at the North American scale, elevation was not a predictor for *Bd* detection, rather species richness, temperature, and biome were [36]. This lends further support that regional and local spatial scales may be more appropriate levels of investigation to assess risk to amphibian populations and species. Species representing these three amphibian families occur throughout our study area, and the effect of the amphibian community assemblage on *Bd* occupancy of their breeding habitats warrants further investigation.

### Water quality as a predictor of detection probability

Our study revealed a potential relationship between pH and *Bd* detection probability. Other water quality covariates that we had predicted could be related to *Bd* occupancy and detection showed no relationships, perhaps because of the scale of our measurements. Specific conductance (conductivity in microSiemens, µS cm^−1^, standardized to 25°C) and turbidity are coarse measurements that indicate the relative amount of dissolved ions and optical properties of the water but do not identify the specific constituents in the water that may be important to *Bd* occupancy and detection (e.g., Ca^+2^, Mg^+2^, Na^+1^, K^+1^, Cl^−1^, nitrogen and phosphorus species, organic carbon, turbidity source, sediment loading, phytoplankton community). The observed temperature and pH ranges at our sites were always within the range that *Bd* grows in the laboratory, and both metrics were often within the range for optimal growth. Our results did not support our prediction that detection probability would be highest when pH was within the range for ideal *Bd* growth [52]. Although the best models included pH and suggested detection probability increased as pH increased, the differences in detection probability were negligible when the pH range was considered (Figure 6). While there may be a narrow pH range that is optimal for *Bd* growth (pH 6–7) in a laboratory setting [52], this narrow range may not be ecologically relevant given the fluctuations in pH at different time scales, such as diel, episodic, and seasonal [101], [102].

pH is influenced by abiotic (e.g., acid-neutralizing capacity) and biotic (e.g., organic carbon, aquatic plant community) characteristics of a system [103]–[105], and it is not clear if pH itself is a mechanism influencing *Bd* detection probability within our study area or if pH is instead reflective of other processes of the aquatic system that are important to *Bd* ecology. Lower pH can inhibit microbial metabolism [106], which could decrease detection probability. Changes in pH are related to the acid-neutralizing capacity in a system, which is strongly tied to the amount of organic carbon present [104],[105], which is in turn an important nutrient for aquatic fungi [107]. Higher pH may indicate higher organic carbon [105], which could increase detection probability. Generally, chytrids decompose organic particulate matter [107], and it is not known if *Bd* uses non-animal organic carbon sources. Diel variation of up to pH 1.8 is not uncommon in mesotrophic and eutrophic aquatic systems, which may be driven by the plant communities present [101], [102]. pH increases with the addition of algae and vascular plants [103]. If *Bd* is associating with plants, perhaps as a secondary host, pH as the predictor of detection probability would be indirect. This result provides direction for future research into how pH influences *Bd* site occupancy and detection probability.

### Management Implications

Our results show that water filtration sampling is a reliable way to assess the occurrence of *Bd* in amphibian habitats when 4–5 samples of a small volume, i.e., 60–600 mL of water, are filtered directly from ponds and wetlands, especially at sites less than 1500 m elevation. *Bd* densities recovered were consistently above the detection limit of the qPCR for a quantitative result (10 genomic equivalents), which affirms water filtration as a viable method for both *Bd* detection and quantification in amphibian habitats. Our results are context-dependent, as found in other studies from the southwestern United States where more samples were necessary to achieve 95% confidence that *Bd* was detected when it was present [80]. Our results suggest that a single visit collecting multiple samples in the spring or summer months may be sufficient (and perhaps more effective) for *Bd* detection from water samples, rather than multiple visits collecting one sample throughout the season, however spatial replicates within the same site visit are important [50]. The number of samples required to detect *Bd* is representative of our area of inference and is not intended to serve as a recommendation for a standard protocol, as regional and site-level variation in *Bd* occupancy may require fewer or additional samples [80].

There are few, if any systems, where species detection is perfect [69]. Imperfect detection (false negatives) may occur because a species is rare or cryptic [108], [109], or alternatively, a false positive report or misidentification may occur [110]–[112]. We underestimated *Bd* occupancy of amphibian habitats within our study area by 14%, which is greater than other observations from a different geographic region that used the same filtration method [80]. These results stress the importance of using methods that account for imperfect detection when evaluating the potential occurrence and distribution of organisms when detection probability is less than 1. The probability of detecting *Bd* in amphibians varies based on life stage, and is highest in adults at low elevations [71]. In temperate areas, lentic-breeding amphibians are often explosive breeders [113], [114], and the likelihood of encountering many adult amphibians is limited to the narrow window of time (hours to days) they are present at a site. While larval amphibians are present at a site for longer time periods, the probability of detecting *Bd* when it is present in them is less than 20% [71]. If the objective of a study is to determine the *Bd* status of a site, sampling the habitat rather than the animals may be the most cost-effective approach in terms of time and resources. Sampling the environment also resolves problems with detection due to differences in capture probability between infected and uninfected individuals related to behavioral changes, however the results are limited to *Bd* occupancy of the aquatic habitat sampled, and inferences to the disease status of an amphibian population, or potential secondary hosts or vectors, cannot be made except by sampling them directly.

By focusing research efforts on understanding the ecology of this fungal pathogen outside of the host, conservation efforts can be more informed and focused to meet the management goals and objectives for species at-risk and common species alike. Patterns in amphibian response to *Bd* are different based on biome [36]. The strategy for assessing *Bd* status at a site may be different according to the ecoregion, and may include sampling the habitat, other species that are present, as well as the amphibians that occur at a site. When water sampling is coupled with amphibian sampling, scientists can begin to understand the relationships between *Bd* occupancy and density in the environment, and the occurrence of disease in amphibian populations.
